# Primary retroperitoneal hydatid cyst, a rare novel differential diagnosis of retroperitoneal masses: A case report

**DOI:** 10.1002/ccr3.6615

**Published:** 2022-11-20

**Authors:** Ali Mehri, Ali Esparham, Reza Rezaei

**Affiliations:** ^1^ Endoscopic and Minimally Invasive Surgery Research Center Mashhad University of Medical Sciences Mashhad Iran; ^2^ Student Research Committee, Faculty of Medicine Mashhad University of Medical Sciences Mashhad Iran; ^3^ Department of Thoracic Surgery, Endoscopic and Minimally Invasive Surgery Research Center Mashhad University of Medical Sciences Mashhad Iran

**Keywords:** abdominal pain, echinococcosis, hydatid cyst

## Abstract

The presence of primary retroperitoneal hydatid cysts is rare, even in endemic areas. The authors report a young man with a retroperitoneal hydatid cyst who underwent total cystectomy. The surgeons should suspect hydatid cysts in case of any abdominal cysts, especially in endemic areas, and avoid any spillage and puncture.

## Abstract 2

Even in endemic locations, primary retroperitoneal hydatid cysts are scarce. However, practitioners, particularly surgeons, should think about hydatid cysts as a possible differential diagnosis for retroperitoneal masses.

## 1 INTRODUCTION

Hydatid cyst is a global zoonotic disease due to *Echinococcus granulosus* infection.^1^ The incidence of hydatid cysts can be 50 per 100,000 person‐years in endemic areas.^2^ The prevalence of hydatid cysts increases with age and it is more prevalent in women.^3^ The most common sites of hydatid cysts in the human body are the liver and lungs (95%).^1^ As there are no specific signs and symptoms related to a hydatid cyst, it is usually diagnosed as a result of an incidental finding on imaging examination following an unrelated problem.^4^ Even in endemic areas, the presence of primary retroperitoneal hydatid cysts is scarce. However, clinicians, especially surgeons, should consider hydatid cysts as an important differential diagnosis of retroperitoneal masses.^5^ Here we report a 22‐year‐old patient with a retroperitoneal hydatid cyst.

## 2 CASE PRESENTATION

**FIGURES 1 and 2:**
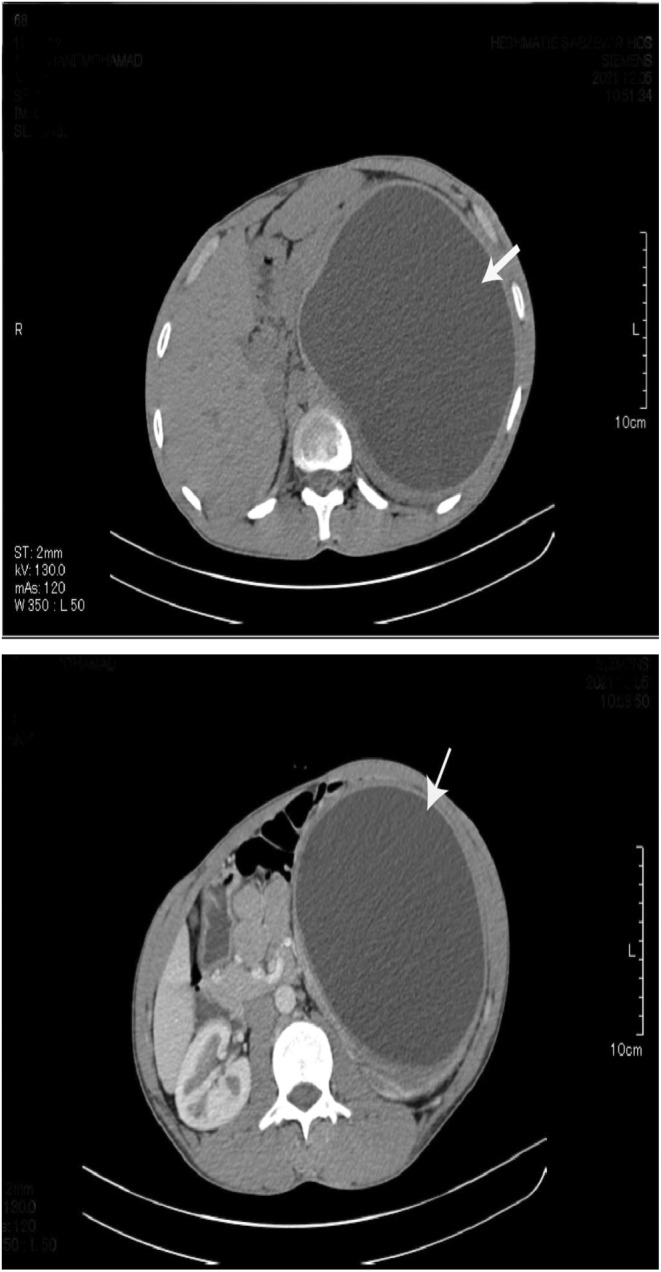
Axial sections of abdomen CT scan without IV contrast

**FIGURES 3 and 4:**
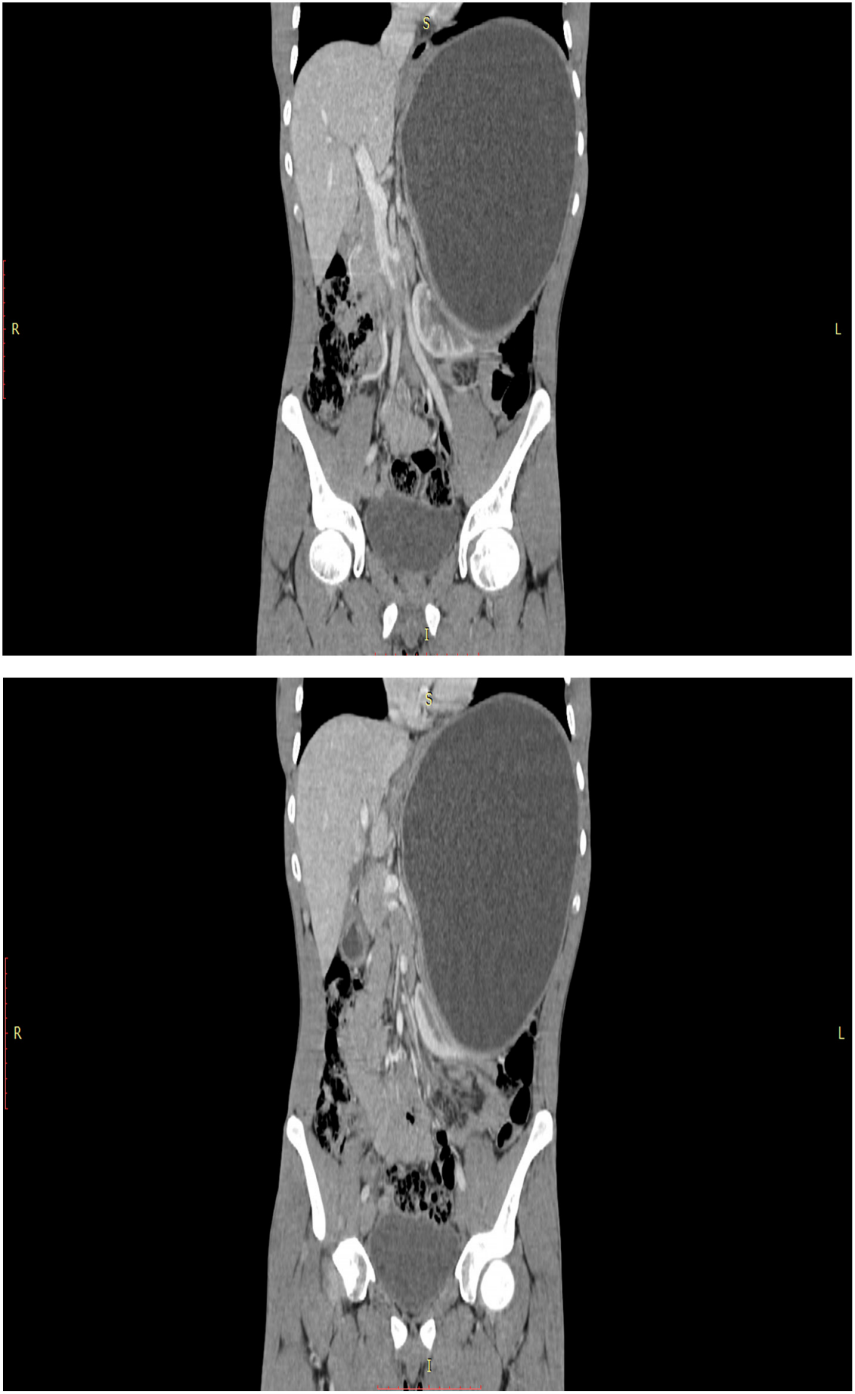
Coronal sections of abdomen CT scan without IV contrast

**FIGURES 5 and 6:**
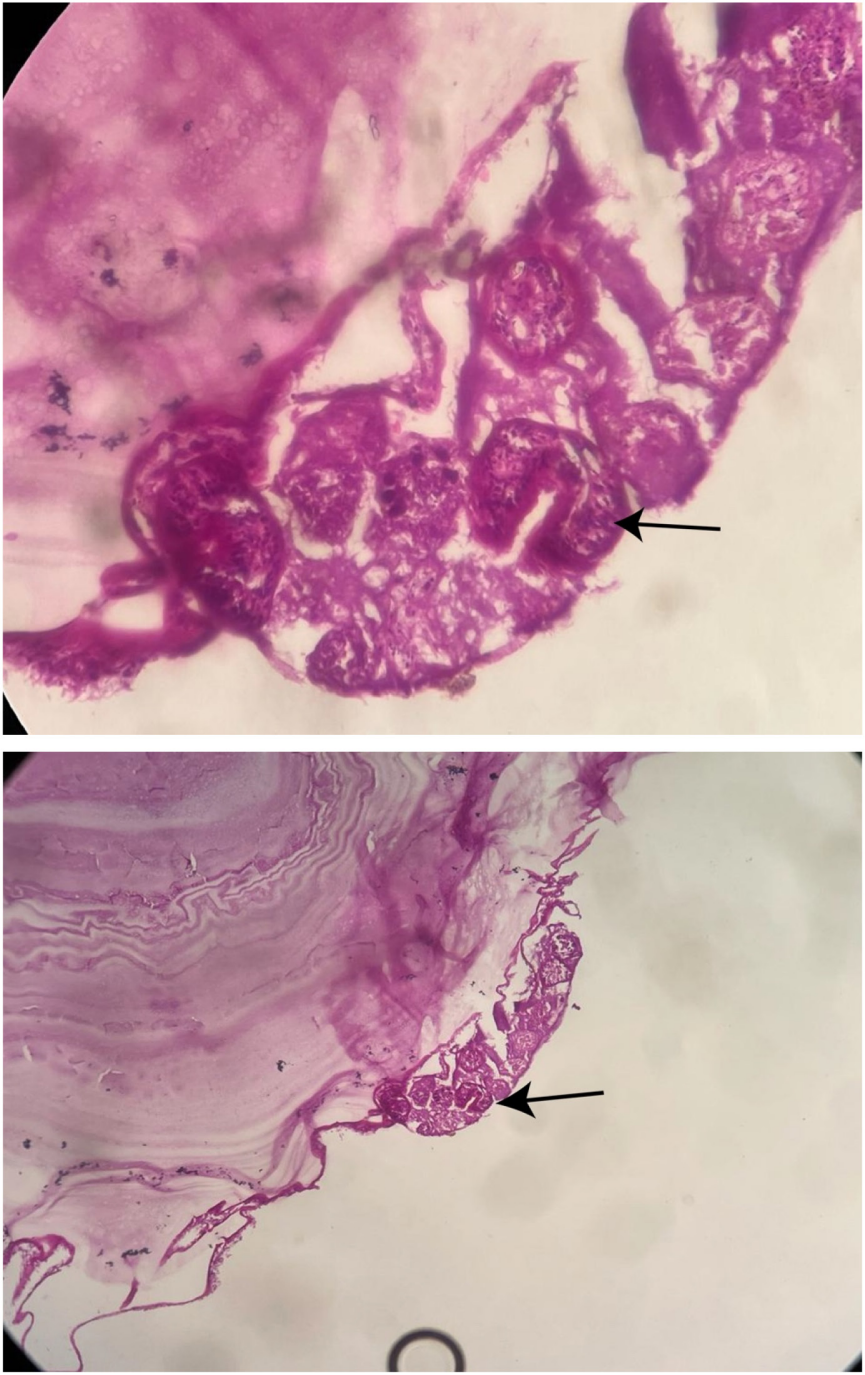
Pathologic images of hydatid cyst wall with scoleces

A 22‐year‐old male patient presented to our center with a chief complaint of local abdominal bulging for at least 12 months. He had no complaint of abdominal pain. The past medical history was insignificant. During physical examinations, a local abdominal bulge was found in the left upper quadrant area. Other systemic physical examinations were normal. All his laboratory investigations were within the normal range. On abdominal ultrasound imaging, an 18 × 13 cm cystic cavity was found in the retroperitoneum area with a hyperechogenic membrane and clear fluid, suggesting a hydatid cyst. Computed tomography showed a vast (20 × 14 cm) retroperitoneal cystic lesion between the diaphragm and bladder, which shifted the abdominal organs to the right side of the abdomen (Figures 1 and 2, 3 and 4). The patient underwent exploratory laparotomy under general anesthesia and in the supine position. A cystic lesion with a laminated membrane was found in the retroperitoneum area extending from the diaphragm to the bladder with a size of 20 × 13 cm. No daughter cyst was found. Enucleation and cystectomy were performed. A tube drain was inserted into the retroperitoneum area. The pathologic examination reported a hydatid cyst (Figures 5 and 6). The patient was discharged after 3 days in good condition. The patient received Albendazole treatment preoperative and also for 6 months after surgery. The clinical follow‐up after 6 months showed no evidence of recurrence, and he was satisfied with the results.

## 3 DISCUSSION

*Echinococcus granulosus* is a 5 mm worm that can live for 5–20 months within the jejunum of dogs. *Echinococcal* eggs exist in the small intestine of canine animals and excrete with feces. Intermediate hosts such as humans, sheep, or cattle ingest these eggs. Then, these eggs penetrate through the intestinal mucosa, enter the blood and lymphatic circulation, and affect body organs.^6^ Humans are accidental hosts for *E. granulosus*. It was stated that *E. granulosus* mostly (95%) affects the lungs and liver, while only 5% involved other organs.^1^ Although retroperitoneal cystic masses are a rare condition, they have different prognoses, and several differential diagnoses should be considered, such as pseudomyxoma retropritonei, cystic lymphangioma, retroperitoneal abscess, soft tissue tumors, and hydatid cyst. The diagnosis can be made by detailed imaging.^7^, ^8^ Even in endemic countries, the presence of primary retroperitoneal hydatid cysts is extremely uncommon.^5^ The retroperitoneal hydatid cyst can be due to the dissemination of protoscoleces from the gastrointestinal tract to the lymphatic system or hematogenous dissemination following liver or lung bypass.^9^ The clinical presentations of the retroperitoneal hydatid cyst vary from asymptomatic to life‐threatening conditions. The symptoms depend on the cysts' number, location, and size.^10^ Also, complications such as rupture, compression, and secondary infection are the most common complications of the hydatid cyst.^11^ Abdominal mass, pain, and nonspecific presentations such as nausea and vomiting are the main clinical features of the hydatid cyst in symptomatic patients.^12^ As in the present case, our patient presented with only abdominal bulging and did not complain of any other symptoms. However, the definite diagnosis of retroperitoneal hydatid cyst depends on the combination of clinical presentation, imaging and pathologic findings, and serologic examination.^9^, ^13^ The imaging modalities for hydatid cysts consist of ultrasound imaging, CT (computerized tomography) scanning, and MRI (magnetic resonance imaging) with high sensitivity. The presence of the daughter cysts, hydatid sands, and the floating membrane can confirm the diagnosis of the hydatid cyst.^14^

Also, in the serologic examination, the detection of Immunoglobulin G antibody by the ELISA (enzyme‐linked immunosorbent assay) method showed high sensitivity and specificity (95% and 94%, respectively).^15^ Several previous studies have reported the presence of hydatid cysts in the retroperitoneal region. Ozturk et al. reported an isolated retroperitoneal hydatid cyst in the splenic hilum area with abdominal pain presentation. Further CT scan findings showed a cystic lesion in the retroperitoneal area, which further pathologic assessment after surgical removal of the cyst revealed a hydatid cyst.^16^ Also, a complicated retroperitoneal hydatid cyst rupture into the intraperitoneal and abdominal wall is reported.^17^ Similar to our case, Sherwani et al.^18^ reported a case of young patients who presented with abdominal bulging in the right lumbar and groin area that further examinations showed primary retroperitoneal hydatid cyst. In addition, Murugesan et al. reported a 45‐year‐old woman who presented with bulging in the right lower quadrant of the abdomen and pain. Exploratory laparotomy revealed a primary retroperitoneal hydatid cyst.^19^ Also, Erdem et al.^9^ described a case of primary retroperitoneal hydatid cyst that presented with dysuria, frequency, urgency, and abdominal pain for 2 months.

The gold standard treatment for hydatid cysts is total cystectomy without contamination.^20^ However, in some cases, when total cystectomy is not possible, partial cystectomy is the best option.^17^ The diagnosis of extrahepatic hydatid cysts is usually tricky. However, surgeons should suspect a hydatid cyst in the case of any abdominal cyst, especially in endemic areas, and avoid spillage and puncture.

## AUTHOR CONTRIBUTIONS

The authors meet the four criteria for authorship based on the International Committee of Medical Journal Editors (ICMJE) recommendations.

## CONFLICT OF INTEREST

All authors declare no conflicts of interest.

## CONSENT

Written informed consent was obtained from the patient to publish this report in accordance with the journal's patient consent policy.

## Data Availability

Data sharing is not applicable to this article as no datasets were generated or analyzed during the current study.
